# Evaluating Antimalarial Proteasome Inhibitors for Efficacy in *Babesia* Blood Stage Cultures

**DOI:** 10.1021/acsomega.4c04564

**Published:** 2024-10-28

**Authors:** Luïse Robbertse, Pavla Fajtová, Pavla Šnebergerová, Marie Jalovecká, Viktoriya Levytska, Elany Barbosa da Silva, Vandna Sharma, Petr Pachl, Jehad Almaliti, Momen Al-Hindy, William H. Gerwick, Evžen Bouřa, Anthony J. O’Donoghue, Daniel Sojka

**Affiliations:** a Institute of Parasitology, Biology Centre of the Czech Academy of Sciences, Ceske Budejovice 370 05, Czech Republic; b Faculty of Science, University of South Bohemia, Ceske Budejovice 370 05, Czech Republic; c Skaggs School of Pharmacy and Pharmaceutical Sciences, University of California San Diego, San Diego, La Jolla, California 92093-0755, United States; d Institute of Organic Chemistry and Biochemistry, Academy of Sciences of the Czech Republic, Prague 117 20, Czech Republic; e Center for Marine Biotechnology and Biomedicine, Scripps Institution of Oceanography, University of California San Diego, San Diego, La Jolla, California 92093-0212, United States

## Abstract

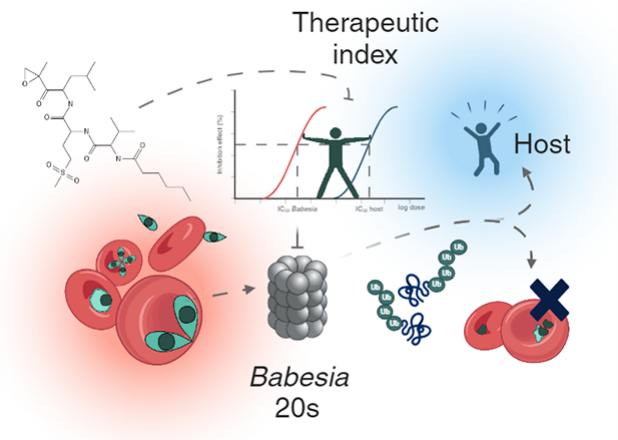

Tick-transmitted *Babesia* are a major global veterinary threat and an emerging risk to humans. Unlike their *Plasmodium* relatives, these erythrocyte-infecting Apicomplexa have been largely overlooked and lack specific treatment. Selective targeting of the *Babesia* proteasome holds promise for drug development. In this study, we screened a library of peptide epoxyketone inhibitors derived from the marine natural product carmaphycin B for their activity against *Babesia*. Several of these compounds showed activity against both the asexual and sexual blood stages of *Plasmodium falciparum*. These compounds inactivate β5 proteasome subunit activity in the lysates of *Babesia divergens* and *Babesia microti* in the low nanomolar range. Several compounds were tested with the purified *B. divergens* proteasome and showed IC_50_ values comparable to carfilzomib, an approved anticancer proteasome inhibitor. They also inhibited *B. divergens* growth in bovine erythrocyte cultures with solid EC_50_ values, but importantly, they appeared less toxic to human cells than carfilzomib. These compounds therefore offer a wider therapeutic window and provide new insights into the development of small proteasome inhibitors as selective drugs for babesiosis.

## Introduction

Babesiosis, recognized as a worldwide emerging malaria-like disease,^1^ is caused by tick-transmitted apicomplexan parasites of the genus *Babesia*, order Piroplasmida—a sister group to Hemosporida that compromises malarial plasmodia.^2^ This disease has a lifecycle comparable to *Plasmodium* species that involves invertebrate vector stages and vertebrate host phases. In the vertebrate hosts, the parasites invade red blood cells, leading to their destruction and symptomatic disease.^3,4^*Babesia* infections have long been recognized as an economically important disease of livestock with growing incidence in both domesticated and wildlife animals.^5^ Bovine babesiosis, commonly called red water fever, is the economically most important arthropod-transmitted pathogen of cattle causing mortalities, abortions, and decreased meat production.^6^ Babesiosis is also gaining attention for its increasing impact on human health. This is evidenced by a rising number of cases expanding geographical ranges of transmission and the inclusion of babesiosis among the CDC’s list of notifiable diseases in 2011, highlighting its emergence as a public health concern.^7,8^

The pathology in humans ranges from clinically silent infections to intense malaria-like episodes representing a fatal risk particularly for elderly or immunocompromised patients.^1^ With a limited number of *Babesia* species known to infect humans—primarily *Babesia microti* in the United States^3^ and *Babesia divergens* in Europe^1^—treatment has traditionally relied on a combination of antimalarial drugs (atovaquone) and antibiotics (azithromycin) in mild cases, while clindamycin and quinine are used in severe cases.^9^ However, the emergence of drug-resistant *Babesia* strains^10^ and recurrent infections in immunocompromised and asplenic individuals^11^ underscores the urgent need for novel therapeutic approaches.^9,12−15^ This need is further compounded by reports of new geographical regions affected and the identification of *Babesia* species as agents of severe human disease, suggesting rapid epidemiological changes.^1,16^ Given the zoonotic nature of babesiosis, which affects both animals and humans, and its transmission dynamics that involve wildlife and environmental factors, the One Health approach interconnecting the health of humans, animals, and our shared environment becomes essential.^17^

Proteasomes are crucial protein complexes found in all eukaryotic cells that play a significant role in degrading proteins marked by ubiquitin.^18^ This core complex is composed of a barrel-shaped structure made from four rings of heptameric proteins. The two outer rings consist of 7 different α subunits, while the two inner rings consist of 7 different β subunits. Within the β rings are three catalytic subunits, namely β1 (caspase-like), β2 (trypsin-like), and β5 (chymotrypsin-like).^19^ Given their role in protein turnover, proteasomes have emerged as promising targets for treating parasitic diseases.^20^ Importantly, the unique structural characteristics of parasite proteasomes allow for inhibitors to be developed that selectively inhibit these complexes without targeting the proteasome of the host cells.^21^ This has sparked interest in developing proteasome inhibitors as potential antimalarial agents^22^ but also as novel drugs to treat leishmaniasis, African sleeping sickness, Chagas disease,^23,24^ trichomoniasis,^25^ and schistomiasis.^26^ Building upon previous work that validated the *Babesia* proteasome as a viable drug target,^27^ this study focuses on evaluating a library of peptide-epoxyketone inhibitors derived from the natural compound carmaphycin B. Compounds in this series are potent against the *Babesia*-related malaria-causing *Plasmodium falciparum* but importantly have low cytotoxicity against mammalian cells.^28^ In this study, we evaluated carmaphycin B derivatives against *B. microti* and *B. divergens*, aiming to identify therapeutics for babesiosis and other piroplasmid infections. This effort contributes to developing innovative interventions against the evolving epidemiological landscapes of tick-borne diseases.

## Materials and Methods

### Parasite Propagation in Bovine Erythrocyte Cultures and Infected Mice

Blood stage cultures of *B. divergens* (strain 2210A G2) were maintained in erythrocytes from defibrinated bovine blood (BioTrading Benelux B.V.). Parasites were multiplied in in vitro erythrocyte cultures containing RPMI 1640 medium (Lonza, cat. no. BE12-115F) supplemented with 50 μg/mL gentamicin (Lonza), 0.25 μg/mL amphotericin B (Sigma-Aldrich), and 20% (v/v) heat-inactivated fetal calf serum (Lonza, inactivation at 56 °C for 30 min before use). Cells were cultured at 37 °C in a 5% CO_2_ environment. *B. microti* (Franca) (ATTC PRA-99) was maintained by continuous intraperitoneal passages in BALB/c female mice (Charles River Laboratories). All laboratory animals were treated in accordance with the Animal Protection Law of the Czech Republic No. 246/1992 Sb., ethics approval no. 25/2018. The study was approved by the Institute of Parasitology, Biology Centre of the Czech Academy of Sciences and the Central Committee for the Animal Welfare, Czech Republic (protocol no. 1/2015).

### Preparation of *Babesia* Lysates

*B. divergens* and *B. microti* protein lysates were prepared from infected red blood cells. Briefly, red blood cells were lysed with 0.15% saponin prepared in 1× Phosphate-Buffered Saline (PBS), pH 7.5, to release intact parasites. Parasites were washed with 1× PBS, pH 7.5 (PBS), by centrifugation and the pellets were stored at −80 °C in PBS plus 20% glycerol. To prepare protein lysates for enzymatic assays, parasites were thawed and transferred to 50 mM HEPES (pH 7.5), 10 μM E64, 100 μM AESBF, 1 μM Pepstatin A, and 1 mM DTT (all Sigma-Aldrich). Cells were ruptured by ultrasonication with an amplitude of 0.5 for 3 cycles of 15 s on ice. To prepare protein lysates for activity-based probing and proteasome purification, parasites were thawed and transferred to 20 mM Tris-HCl, pH 7.5, 1 mM DTT, 10 μM E64, and 5% glycerol. The lysate was sonicated on ice for 3 times and clarified by centrifugation at 17,000*g* for 10 min at 4 °C.

### Enrichment of *B. divergens* 20S Proteasome

The clarified supernatant was centrifuged in a Beckman Coulter Op-80 XP ultracentrifuge at 82,656*g* for 30 min to remove cellular debris, mitochondria, and other organelles. Subsequently, a 3.5 h ultracentrifugation step at 277,816*g* was performed to pellet the 20S proteasomes, while smaller cell matrix proteins remained in the supernatant. The resulting pellet was resuspended in 20 mM Tris-HCl buffer, pH 7.5, and loaded onto an anion-exchange HiTrap MonoQ HP column. Elution was achieved using a gradient of 1 M NaCl. Fractions containing *B. divergens* 20S (Bd20S) proteasome were identified through enzyme activity assay, pooled, concentrated (100 kDa, Merck Millipore), and further purified by gel filtration using a Superose 6 Increase column (GE Healthcare) equilibrated with 20 mM Tris-HCl, pH 7.5. Fractions sensitive to bortezomib were pooled, concentrated, analyzed using NuPAGE Tris-Acetate protein gels (Invitrogen, Thermo Fisher), and stored at −80 °C. After each chromatographic step, the activity of all fractions was assayed with 50 μM substrate succinyl-Leu-Leu-Val-Tyr-aminocoumarin (Suc-LLVY-amc) in assay buffer (50 mM HEPES, pH 7.5, and 1 mM DTT), along with 100 nM recombinant human PA28α with 10 μM bortezomib or DMSO as a control. The release of AMC fluorophore was monitored at Ex 340 nm/Em 460 nm at 37 °C using a Synergy HTX multimode reader (BioTek).

### Optimization of Enzyme Activity

To further improve the detection of the β5 subunit activity with the Suc-LLVY-amc substrate of the *Babesia* proteasome as previously published,^27^ we aimed to further optimize assay conditions. However, to reduce the number of experimental animals in the study, assay conditions were optimized using protein lysates of *B. divergens*, which can be grown *in vitro,* instead of *B. microti* lysates, which need to be maintained in mice. However, the optimized assay conditions were then confirmed to work for *B. microti* lysates. Assay conditions were optimized in 50 μL volumes in round-bottom 96-well plates. Two assay buffers were evaluated. Buffer A, as described previously,^27^ consists of 20 mM HEPES, pH 8, 1 mM ATP, and 1 μM E64, while Buffer B consists of 50 mM HEPES, pH 7.5, 5 mM ATP, 10 μM E64, 100 μM AESBF, 1 μM Pepstatin A, and 1 mM DTT. Buffers A and B were tested with either 0.02% SDS or 6.67 ng of PA28 as activators of the proteasome activity. After confirming Buffer B (with addition of PA28α (BioTechne #E-381)) as a suitable buffer, two other activator subunits were tested in Buffer B, including PA28β (BioTechne #E-382) and PA28γ (BioTechne #E-384). The optimized assay conditions were then confirmed using *B. microti* lysates. The release of the AMC fluorophore was monitored at 37 °C in a Synergy HTX multimode reader (BioTek) with excitation and emission wavelengths set to 340 and 460 nm, respectively.

### Recombinant Expression and Purification of PA28α

As PA28α was found to activate Bd20S, we expressed this enzyme in *Escherichia coli*. The codon-optimized gene (Uniprot ID Q06323) encoding human PA28α protein was commercially synthesized (GeneArt, Thermo Fisher) and cloned into a pSUMO vector, which encodes an N-terminal His8×-SUMO tag. *E. coli* Bl21 (DE3) cells were transformed with the expression vector and grown at 37 °C in LB medium. Once the OD_600_ nm reached 0.5, protein expression was induced by adding IPTG to a final concentration of 500 μM, and the protein was expressed overnight at 16 °C. Bacterial cells were harvested by centrifugation, resuspended in lysis buffer (50 mM Tris-HCl, pH 7.5, 5 mM MgSO_4_, 150 mM NaCl, and 1 mM DTT), and lysed by sonication. The lysate was then cleared by centrifugation. Subsequently, the supernatant was loaded onto a HisTrap HP column (Cytiva) and washed with lysis buffer supplemented with 20 mM imidazole, and the protein was eluted with lysis buffer supplemented with 250 mM imidazole. The protein was buffer-exchanged (using a 10 kDa cutoff concentrator, Merck Millipore) into lysis buffer and digested with Ulp1 SUMO protease (Invitrogen, Thermo Fisher) at 4 °C overnight. The SUMO tag was removed by a second incubation with the HisTrap HP column. The purified protein was concentrated to 56 μM, aliquoted, and stored at −80 °C until needed.

### Bd20S Activity and Inhibition Assays

To assess the enzyme activity, 8 ng of purified preparation was preincubated with 0 to 4000 nM recombinant human PA28α for 1 h at room temperature (RT). The enzyme activity was then assayed with 50 μM Suc-LLVY-amc in 50 mM HEPES, pH 7.5, and 1 mM DTT in the presence of 250 nM PA28α. To evaluate the substrate specificity for β5, β2, and β1 subunits, a set of available proteasome substrates was screened that included Suc-LLVY-amc, Z-VLR-amc and Z-LLE-amc, Ac-FnKL-amc, Ac-nPnD-amc, Ac-RYFD-amc, Ac-FRSR-amc, and Ac-GWYL-amc. Bd20S was initially activated with PA28α and the rate of release of 7-amino-4-methyl coumarin (amc) was quantified over time following addition of the substrates. For control, 6 μM marizomib (MZB, MedChemExpress) was used to ensure substrate cleavage by proteasome. For inhibition studies, IC_50_ values were measured using presteady-state enzyme kinetics. Five or 100 nL of inhibitor at concentrations ranging from 10 mM to 170 nM was dispensed using the ECHO 650 liquid handler (Beckman). Subsequently, 4 μL of 100 μM substrate was added, followed by 4 μL of Bd20S, and the release of amc was immediately measured. The IC_50_ values were calculated from the fluorescence units per second (RFU/s) recorded between 60 and 90 min after the reaction. All assays were performed in black 384-well plates, with a final assay volume of 8 μL, and each condition was run in triplicate technical replicates. Data were analyzed and plotted using GraphPad software.

### SDS-PAGE and Activity-Based Probing

Twelve micrograms of total *B. divergens* protein lysate was diluted in 50 mM HEPES, pH 7.5, containing 10 μM E64 and mixed with a selected inhibitor at a final concentration of 5 μM or a vehicle control. After 1 h preincubation, the activity-based probe Me4BodipyFL-Ahx3Leu3VS (R&D Systems #I-190) was added to reach its final concentration of 2 μM. The samples were incubated at RT for 16 h. For SDS-PAGE, samples were mixed with NuPAGE LDS Sample Buffer (4×; Invitrogen, Thermo Fisher) containing 100 μM dithiothreitol, heated at 100 °C for 10 min, and loaded onto a NuPAGE 12% Bis-Tris gel (Invitrogen, Thermo Fisher). Gels were run in 1× MOPS SDS buffer (Invitrogen, Thermo Fisher) at 130 V. The gel was scanned on Bio-Rad ChemiDoc MP using the Alexa 488 program and subsequently stained in Coomassie brilliant blue. The protein load was visualized using Bio-Rad ChemiDoc MP.

### Western Blot Analysis

To assess the on-target effect of proteasome inhibition, protein lysates of *B. divergens* isolated from bovine erythrocyte cultures were evaluated for the accumulation of polyubiquitinated proteins. The *B. divergens* cultures underwent two 12 h treatments (totaling 24 h) with 200 nM of the inhibitor in culture medium. Parasites were released from RBCs and protein lysates were prepared as described above. The resulting samples were then centrifuged at 15,000*g* for 15 min at 4 °C and protein concentrations of the resulting supernatant were determined using Bradford assay.^29^ Samples were prepared for SDS-PAGE in reducing Laemmli buffer supplemented with β-mercaptoethanol. Ten micrograms of protein was applied per lane and proteins were separated on gradient (4–15%) Criterion TGX Stain-Free Precast gels (BioRad) in Tris-Glycine-SDS running buffer (25 mM Tris, 192 mM glycine, and 0.1% (w/vol) SDS, pH 8.3) and visualized using TGX stain-free chemistry (BioRad). Proteins were then transferred onto a PVDF (polyvinylidene difluoride) membrane using a Trans-Blot Turbo system (BioRad) and membranes were blocked in 3% (w/v) nonfat skimmed milk in 1× PBS with 0.05% Tween 20 (PBS-T; Sigma-Aldrich). For immunostaining, membranes were incubated overnight at 4 °C in a 1:2000 dilution of Anti-Ubiquitin K48 Linkage antibodies (Boston Biochem) in milk-PBS-T, washed (3 × 5 min) in PBS-T, and subsequently exposed to the goat antirabbit IgG-peroxidase secondary antibody (Sigma) diluted by 1:2000 in milk-PBS-T at room temperature for 1 h. After washing (3 × 10 min) in PBS-T, signals were detected using Clarity Max Western ECL substrate (BioRad), visualized using a ChemiDoc MP imager, analyzed, and adjusted using the ImageLab freeware (BioRad).

### Activity Assays with *Babesia* Cell Lysates

Proteasome activity in *B. microti* and *B. divergens* lysates was screened in the presence of carmaphycin B (CPB) and numerous analogs that were previously designed to target the *P. falciparum* 20S proteasome.^30^ A pool of lysates was made for each species to ensure that equal amounts of the lysates were used in each inhibition reaction. Inhibitors were transferred to black 384-well plates with the ATS acoustic transfer system (ATS-100, Biosera). Lysates were incubated with inhibitors for 30 min at room temperature and activity was assayed in 30 μL reactions. Each reaction mixture contained 5 μL of *Babesia* lysate, 10 nL of the inhibitor (final concentration of 133.33 nM), and 25 μL of assay buffer with 25 μM substrate succinyl-Leu-Leu-Val-Tyr-7-amido-4-methylcoumarin (Suc-LLVY-amc). The release of the AMC fluorophore was monitored at 37 °C in a Synergy HTX multimode reader (BioTek Instruments, Winooski, VT) with excitation and emission wavelengths set to 340 and 460 nm, respectively. Activity was quantified as RFU per second and normalized to the DMSO control reactions.

### Compound Effectivity in Erythrocyte Cultures Infected with *B. divergens* (In Vitro)

To evaluate the effect of inhibitors on *B. divergens* replication, cultures (5% hematocrit) containing 2% parasitemia were cultivated in media. Single concentration screens were first performed using 600 nM of compound and then dose–response assays (5400 to 2.34 nM) were subsequently performed for compounds of interest. Assays were performed twice in triplicate wells in a 96-well tissue culture plate format (TPP, Switzerland) and media containing inhibitors were replaced at 12 h intervals for a total of 48 h. DMSO diluted in medium (0.1%) served as a vehicle control. The parasitemia of intraerythrocytic *B. divergens* was determined using flow cytometry following the staining of nucleic acids as described previously.^31^ Briefly, infected red blood cells were fixed in a solution of 4% paraformaldehyde and 0.025% glutaraldehyde in PBS for 30 min at room temperature (RT). The fixed cells were subsequently washed twice with 1× PBS (600*g*, 3 min) and stained with 0.02 mM Ethidium Homodimer 1 (EthD-1, Biotinum) diluted in PBS for 30 min at RT. Parasitemia was analyzed using a FACS CantoII flow cytometer and Diva software provided by BD Biosciences. The EC_50_ of compounds in the dose–response inhibition was determined in GraphPad Prism 10 using nonlinear regression.

### Protein Alignments and Phylogenetic Analysis

A multiple alignment of proteasome was performed on the Clustal Omega program (Clustal O (1.2.4)) using default parameters. The resulting alignment was visualized using ESP3 software.^32^ The phylogenetic analysis of the multiple alignment was performed using the maximum likelihood method in IQ-TREE.^33^ The tree was visualized using the FigTree 1.4.4. program.

### Molecular Modeling

For molecular modeling, we generated structures of the catalytic core particle of *B. divergens* and *B. microti* proteasomes using the SWISS-MODEL homology modeling server. Whole structures were imported into Schrödinger suite (Schrödinger, LLC, New York, NY, 2024) and prepared for further modeling by the Prime module. All compounds were docked into the active site of β5 subunit defined by the homology structure—complex of yeast 20S proteasome with covalently bound carmaphycin A (PDB 4HRD).^34^ Compounds were docked using CovDock (Schrödinger, LLC, New York, NY, 2024), and figures were generated by Schrödinger gui Maestro.

## Results and Discussion

In this study, we built on our previous efforts to explore selective proteasome inhibition as a potential new treatment approach for babesiosis.^27^ Given the close evolutionary relationship of *Babesia* species with malaria-causing parasites, we tested the marine cyanobacterial metabolite carmaphycin B. This compound is known for its potency against both the asexual and sexual stages of *P. falciparum*(28) and was chosen as the base molecule for our investigation. Carmaphycin B is a tripeptide-epoxyketone, which is similar in structure to the tetrapeptide-epoxyketone inhibitors epoxomicin and carfilzomib, which we and others have previously shown to kill *B. divergens* in the low nanomolar range.^27,35^ Although carmaphycin B effectively targets the *P. falciparum* proteasome, it also poses significant toxicity risks to mammalian cells. Consequently, we explored derivatives of carmaphycin B that maintain high effectiveness against the parasite while reducing toxicity to the host, thus improving the selectivity index—the measure of drug efficacy between the parasite and host proteasomes.^28,30^ However, to improve the readability of our assays, we had first to optimize enzymatic assays involving *Babesia* lysates and to enrich for the *B. divergens* proteasome.

### 20S Proteasome of *B. divergens* and *B. microti*

**Figure 1:**
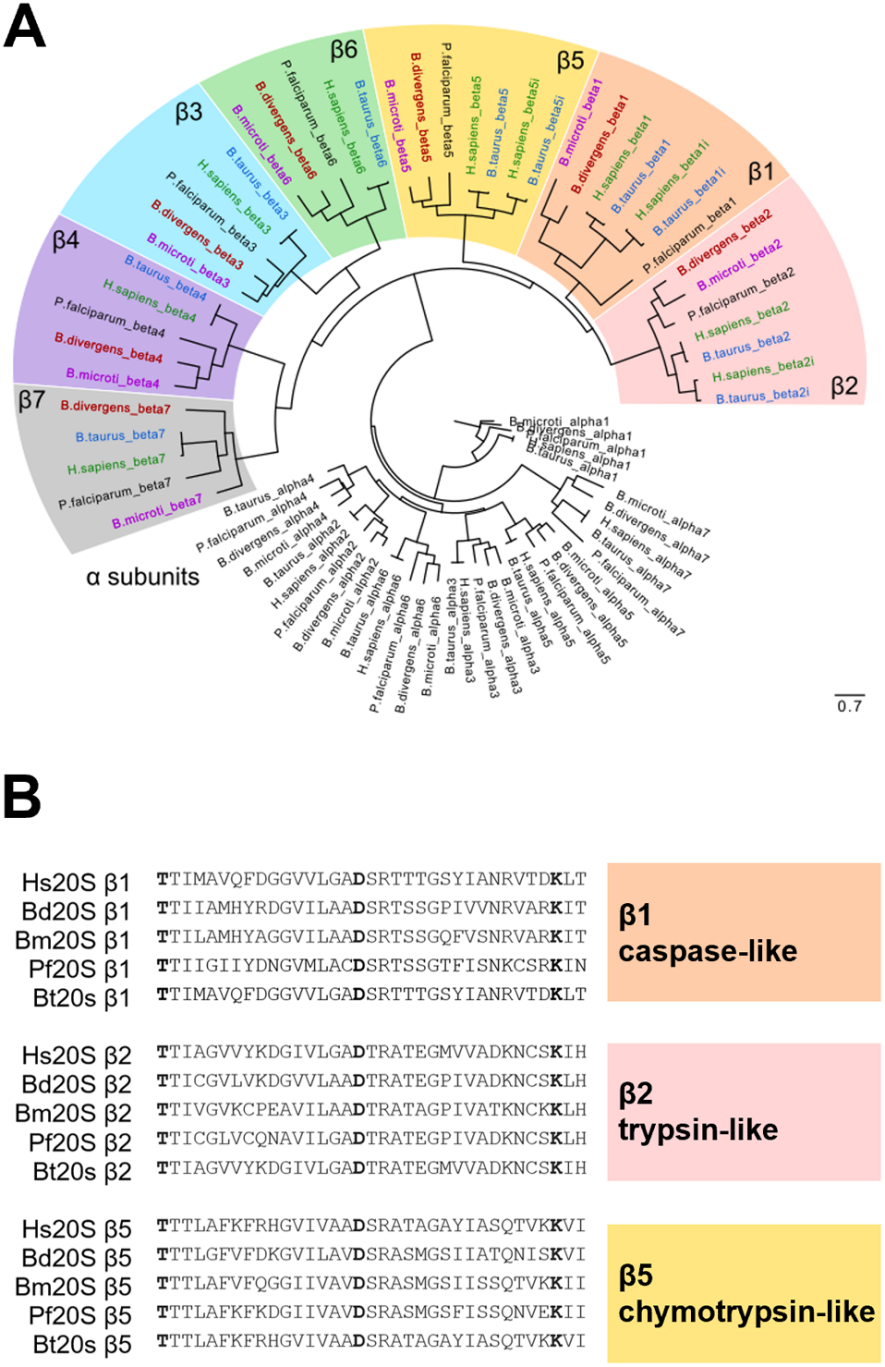
Phylogenetic analysis and proteasome subunit identification in *B. microti* and *B. divergens*. (A) Maximum-likelihood phylogenetic tree displaying the α and β subunits of the 20S proteasome identified in *B. microti* and *B. divergens* clustering with their bovine, human, and *P. falciparum* analogs. Sequence hits for each species are differentiated using color coding for comparative analysis. (B) Detailed alignment of the threonine protease catalytic subunits (residues 1–35) across the β1, β2, and β5 subunits of the 20S proteasomes from human (Hs20S), *B. divergens*, *B. microti*, *P. falciparum* (Pf20S), and *Bos taurus* (Bt20S). Key catalytic residues 1 (Thr), 17 (Asp), and 33 (Lys) are depicted in bold.

The catalytic core of the *Babesi*a proteasome is, as in most other eukaryotes, represented by a cylindrically shaped, large complex of proteins responsible for degrading proteins marked for destruction with polyubiquitin chains, thus controlling numerous cellular processes.^36^ The identification of seven α and seven β 20S proteasome subunits in *B. microti* has been previously reported.^37^ Building upon this work, our study identifies all α and β subunits of *B. divergens*, presenting the complete 20S proteasomes of the two human-affecting species: “sensu lato” *B. microti* and “sensu stricto” *B. divergens*. These species occupy distinct positions in the evolutionary tree of the apicomplexan parasite order Piroplasmida^38^ and serve as laboratory models for subsequent experiments in our studies.^27^ The entire set of identified encoding sequences, listed in Supplementary Table 1, was used to construct a maximum-likelihood phylogenetic tree via the online open-source software IQ-TREE.^33^ This tree clearly confirms the clustering-based identity of each identified sequence, including those of bovine, human, and *P. falciparum* analogs (Figure 1A). Furthermore, Figure 1B illustrates the detailed protein alignment of the threonine protease catalytic subunits—residues 1–35—of the β1, β2, and β5 subunits from the human constitutive 20S proteasome (Hs20S), in addition to the *B. divergens*, *B. microti*, and *P. falciparum* 20S proteasomes. This diversity among the active site pockets within each species and the mammalian host offers a promising avenue for testing existing malaria 20S proteasome selective inhibitors. Additionally, it underscores the potential for developing novel *Babesia*-selective inhibitors with limited toxicity to the host.

### PA28α Activator Increases the Activity of *Babesia* Proteasome β5 Subunit

We and others have been successful in enriching proteasomes from parasite protein lysates^22,25,39^ and have used these enzymes for biochemical and enzymatic assays. However, the purification procedures require milligram amounts of starting material and are generally reagent- and labor-intensive. Therefore, in this study, we focused our efforts on improving the detection of proteasome activity in cell lysates so that many of the key biochemical studies on *Babesia* proteasome could be performed without the need to purify the enzyme.

**Figure 2:**
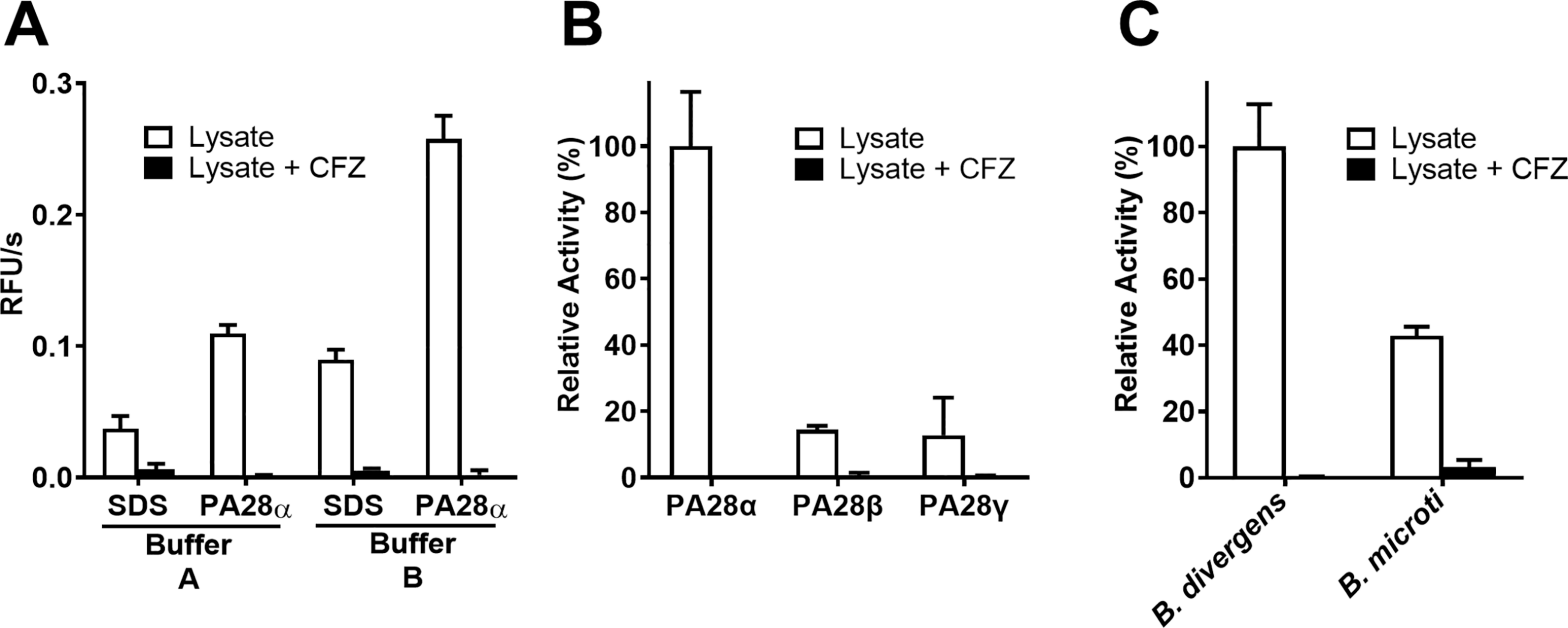
Optimization of fluorescent peptidyl-substrate assay conditions for the β5 proteasome subunit activity in the lysates of *B. divergens* and *B. microti*. (A) Comparison of *B. divergens* activity determined in kinetic assays (shown with an increase in RFU/s) using the Suc-LLVY-amc substrate. Buffer A consists of 20 mM HEPES (pH 8.0), 1 mM ATP, and 1 μM E64 with either 0.02% SDS^27^ or 6.67 ng of PA28α per reaction. Buffer B consists of 50 mM HEPES (pH 7.5), 5 mM ATP, 10 μM E64, 100 μM AESBF, 1 μM Pepstatin A, and 1 mM DTT with either 0.02% SDS or 6.67 ng of PA28α per reaction. (B) Comparison of three different human activators (PA28α, PA28β, and PA28γ) with *B. divergens* lysates in Buffer B (see panel A). (C) Confirmation of chymotrypsin-like activity for the *B. microti* β5 subunit with the optimized assay conditions (Buffer B with PA28α). All activities were inhibited by carfilzomib (CFZ).

To enhance detection of *B. divergens* proteasome activity in cell lysates, we focused on optimizing the assay buffer conditions to promote increased enzymatic activity. In our previous studies, we used an assay buffer consisting of HEPES, pH 8.0, and 0.02% SDS as this low amount of detergent was shown by others^40^ to partially denature the α-ring and therefore open the proteasome to allow substrate access. However, for *Plasmodium* proteasome assays, we and others have shown that the addition of human PA28α increases activity^22,41^ more than SDS. Therefore, we added either SDS or PA28α to the *B. divergens* lysates and directly compared the enzyme activity using the β5 substrate, Suc-LLVY-amc. We showed that PA28α increased enzyme activity by 3-fold, therefore revealing that the human proteasome activator can bind and activate *B. divergens* proteasome (Figure 2A). Importantly, we also showed that this increased activity is due to proteasome as the addition of carfilzomib decreased enzyme activity by >95%. We then did some further optimization of the assay buffer and determined that HEPES, pH 7.5, and the addition of 1 mM DTT (Buffer B) resulted in a further increase in proteasome activity.

Intrigued by the effect of human PA28α on the function of *B. divergens*, we then evaluated two other PA28 structures, namely PA28β and PA28γ (alignment in Supplementary Figure 1). However, proteasome activity in the presence of these other proteins was only 14.41 and 12.83% of the activity with PA28α, respectively (Figure 2B). Finally, we showed that the new assay buffer conditions could be used to detect proteasome activity in *B. microti* lysates (Figure 2C).

Overall, these studies revealed that proteasome inhibition assays can be performed using *B. divergens* and *B. microti* cell lysates and, therefore, compounds can be evaluated for proteasome inactivation without the need for isolating the enzyme.

### Carmaphycin B Is a Potent *Babesia* 20S Proteasome Inhibitor

**Figure 3:**
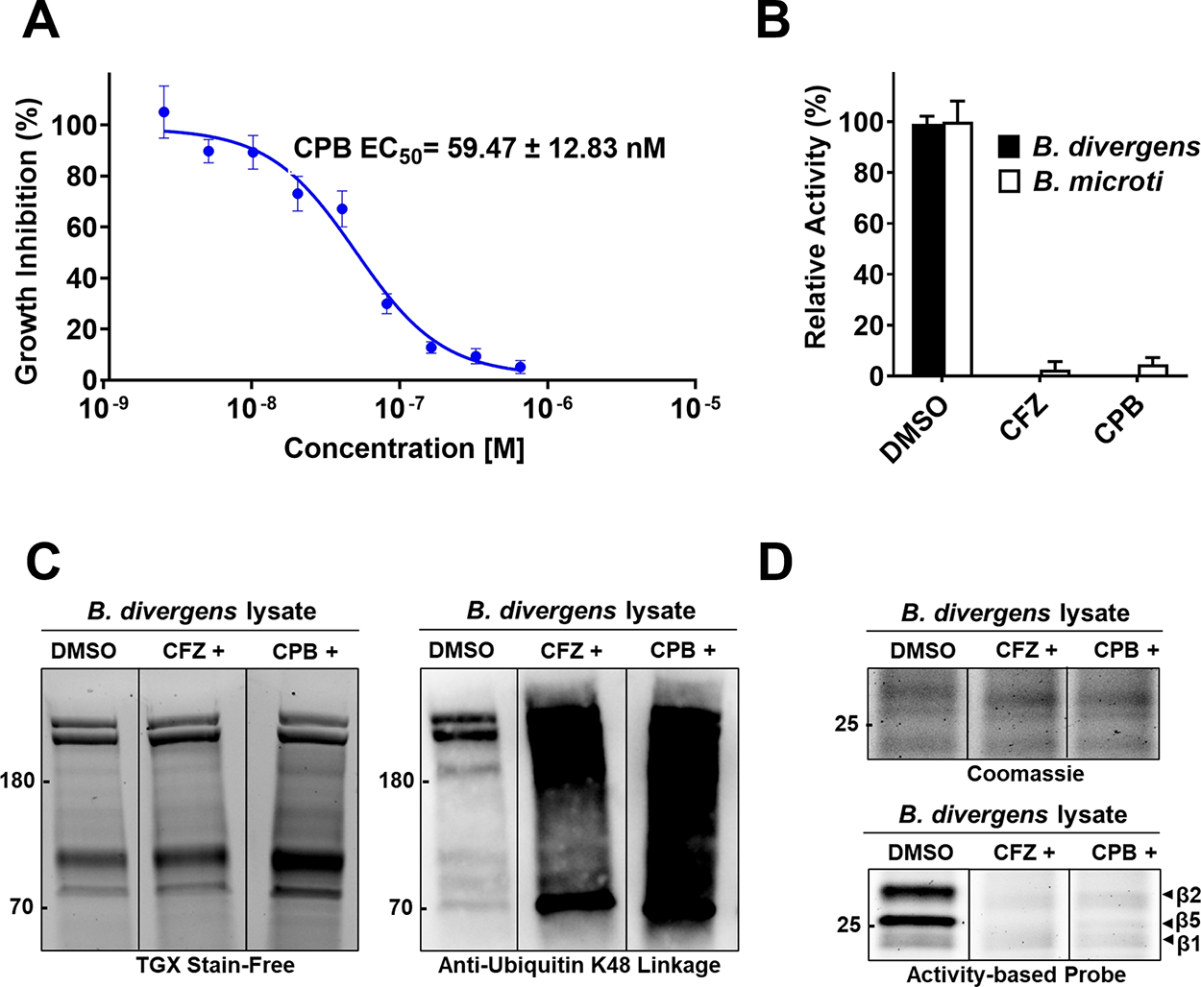
Determination of the potential of a naturally derived proteasome inhibitor to inhibit *Babesia* proteasome. (A) Treatment of *B. divergens* in RBC in vitro cultures with carmaphycin B (CPB). A decrease in *B. divergens* parasitemia is shown with an increasing concentration of CPB. Data represent means of three biological replicates and the error bars indicate standard deviations. (B) Inhibition of β5 *B. divergens* and *B. microti* proteasome activity with 133.33 nM carmaphycin B (CPB) measured using Suc-LLVY-amc. Relative activity (% RFU/s) is calculated using 0.5% DMSO. (C) Accumulation of endogenous polyubiquitinated proteins in *B. divergens* after treatment for 24 h with 200 nM carfilzomib (CFZ+) and carmaphycin B (CPB+). The left gel shows the loading control and the right gel shows the accumulation of polyubiquitinated proteins. (D) Gel-based characterization of Bd20S using Me4BodipyFL-Ahx3Leu3VS activity-based proteasome labeling. The upper gel is the protein loading control and the bottom gel shows the labeling of the catalytic subunits.

Carmaphycin B (CPB) is a tripeptide molecule possessing an *N*-hexanoyl group at the amino end and an α,β-epoxyketone group at the carboxyl end that was extracted from the cyanobacterium *Symploca* sp., collected from Curaçao.^42^ This compound exhibits potent cytotoxic effects against human lung adenocarcinoma and colon cancer cell lines via inhibition of the β5 subunit of the constitutive proteasome. We have previously evaluated three other peptide epoxyketone inhibitors, namely carfilzomib (CFZ), epoxomicin, and ONX-0914, and determined that each of them can significantly reduce *B. divergens* growth in bovine erythrocyte cultures in the 25–200 nM range.^27^ CFZ was the most potent compound with an EC_50_ of 29 nM. We therefore incubated *B. divergens* cultures with carmaphycin B over a concentration range of 2.34–600 nM and calculated an EC_50_ of 59.5 ± 12.83 nM (Figure 3A and Supplementary Figure 2). These data indicate that CPB targets the β5 subunit of Bd20S, which may mediate killing of the parasite. In addition, carmaphycin B decreases β5 activity in *B. divergens* and *B. microti* lysates to the same extent as CFZ (Figure 3B), therefore revealing a similar mechanism of action.

We next evaluated if CFZ and CPB target the proteasome in a live cell. To do this, *B. divergens* in vitro cultures were incubated with 200 nM CFZ and CPB for 24 h before the cells were washed and lysed and the protein lysate was evaluated by western blot using anti-Ubiquitin K48 antibodies. When compared to a vehicle-treated control (0.1% DMSO), treatment with either CFZ or CPB led to a marked accumulation of endogenous polyubiquitinated proteins. This accumulation is indicative of proteasome inhibition as the enzyme is unable to break down polyubiquitinated proteins (Figure 3C). This method indirectly revealed that CFZ and CPB affect protein turnover by the proteasome. To directly show that the compounds engage with the catalytic subunits of Bd20S, we incubated the cell lysates from the CFZ- and CPB-treated cells with an activity-based probe that covalently labels one or more catalytic subunits of proteasomes (Figure 3D). This probe consists of the tripeptide LLL with a C-terminal vinyl sulfone group and an N-terminal BODIPY FL fluorescent reporter group. In the lysate from the vehicle-treated cells, the probe strongly labeled two subunits, which by molecular weight were predicted to be β2 (upper) and β5 (middle). The lower band was weakly labeled, and it was predicted to be β1. When the probe was added to cell lysates from cells that had been treated with CFZ and CPB, the labeling was significantly reduced. These data confirm that CFZ and CPB were able to enter the live cells, directly engage the catalytic subunits of the proteasome, and therefore prevent the subsequent binding by the probe.

### Screening of Carmaphycin Analogs against *B. divergens* and *B. microti* Lysates

**Figure 4:**
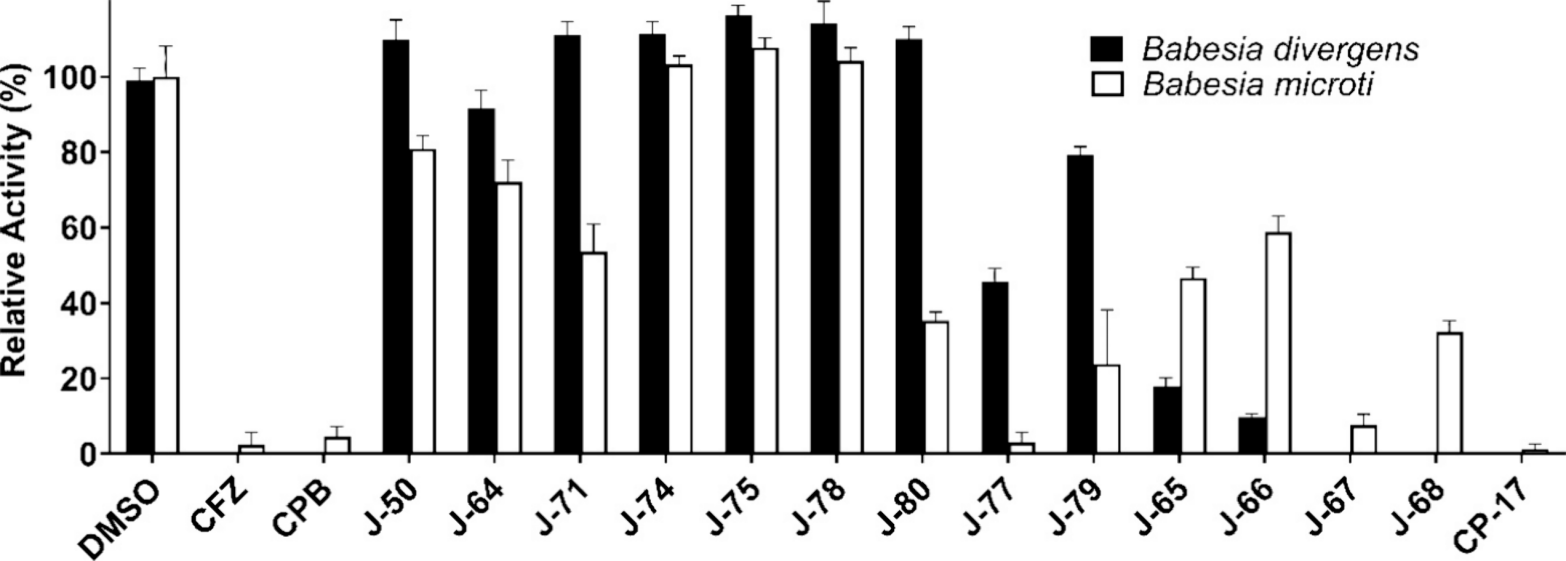
Inhibition of β5 *B. divergens* and *B. microti* proteasome activity by derivatives of carmaphycin B measured in optimized Suc-LLVY-amc fluorescent kinetic assays. Relative activity (% RFU/s) of *B. divergens* and *B. microti* β5 (Suc-LLVY-amc) subunit relative to DMSO control.

We showed that CFZ and CPB can completely inactivate cleavage of Suc-LLVY-amc in *B. divergens* cell lysates and, therefore, this same lysate can be used to screen for new inhibitors of Bd20S. We screened a library of CPB analogs that were designed in a medicinal chemistry effort to target the 20S proteasome of *P. falciparum* of which many have lower potency against the human constitutive proteasome than CPB.^28,30^ In parallel, we screened the same library against the *B. microti* lysate. The enzyme activity in both lysates was inhibited by CFZ and CPB (Figure 4), revealing that they can be used to screen for proteasome inhibitors.

**Figure 5:**
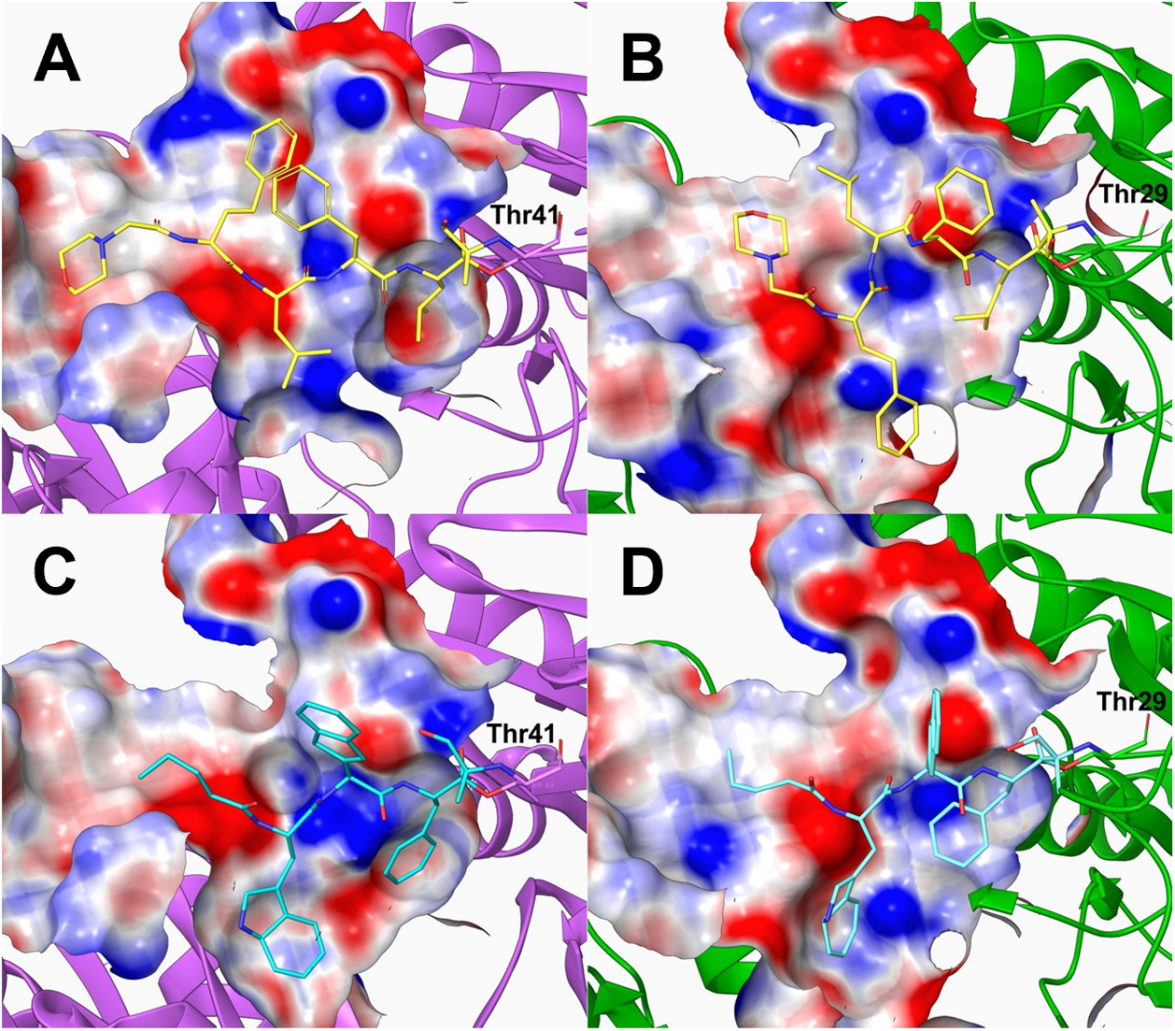
In silico visualization of binding of the maternal CPB (A, B) and CP-17 (C, D) to the *B. microti* (magenta backbone) and *B. divergens* (green backbone) 20S β5 proteasome subunits.

Following preincubation of each compound with *B. divergens* and *B. microti* lysates, we calculated the relative activity to a DMSO-treated sample. We first looked at a selection of compounds that were previously shown to be potent *P. falciparum* inhibitors (EC_50_ < 100 nM) while also having low HepG2 cell cytotoxicity (EC_50_ > 4 μM). These include J-50, J-64, J-71, J-74, J-75, J-78, and J-80 (Figure 4). A common feature of each of these inhibitors is that they contain a d-amino acid in the P3 position, which was highly favorable for binding to the β5 subunit of *P. falciparum* 20S proteasome and unfavored by β5 subunit of the human constitutive proteasome.^28^ It is clear from this screen that the inhibitors in this series did not target the β5 subunit of Bd20S. Bm20S was found to be inhibited by some of these compounds but completely uninhibited by others. An ideal hit compound should be potent against both *B. divergens* and *B. microti* and, therefore, we evaluated other analogs of CPB that did not show good potency or selectivity in the *Plasmodium* studies and were therefore not pursued further as potential antimalarial hits. We clustered these compounds into three groups based on structural similarities. Group 1 consists of J-77 and J-79 that contain l-*N*,*N*-Diethyl-Asn in the P3 position. These compounds differ to J-78 and J-80 with just the change in stereochemistry at the P3 position, but it is clear that *Babesia* proteasomes have a higher preference for l-*N*,*N*-Diethyl-Asn over d-*N*,*N*-Diethyl-Asn. Compounds in Group 2 have either a d-4-pyridyl-Ala (J-64), d-3-pyridyl-Ala (J-65 and J-66), or d-2-pyridyl-Ala (J-67 and J-68) in the P3 position. It is clear that d-2-pyridyl-Ala is preferred by *B. divergens* and *B. microti* over d-3-pyridyl-Ala, while d-4-pyridyl-Ala is unfavored. Finally, Group 3 contains a single compound, CP-17, that was developed in an earlier medicinal chemistry effort for *Plasmodium* proteasome inhibitors.^28^ This compound contains l-Trp in the P3 and P2 positions and had a selectivity index of 37 for *P. falciparum* over HepG2 cells. This compound was also found to be a hit against *Schistosoma mansoni*(26) and *Trichomonas vaginalis*.^39^ In this current study, we also found that CP-17 was the most potent inhibitor of both proteasomes from the two evolutionary distant *Babesia* species (Figure 5). Importantly, this compound has 27-fold lower toxicity for HepG2 cells when compared to CPB and is therefore an ideal starting point for medicinal chemistry efforts.

### J-68 Is the Leading Compound in Selectivity for RBC-Cultured *B. divergens*

**Figure 6:**
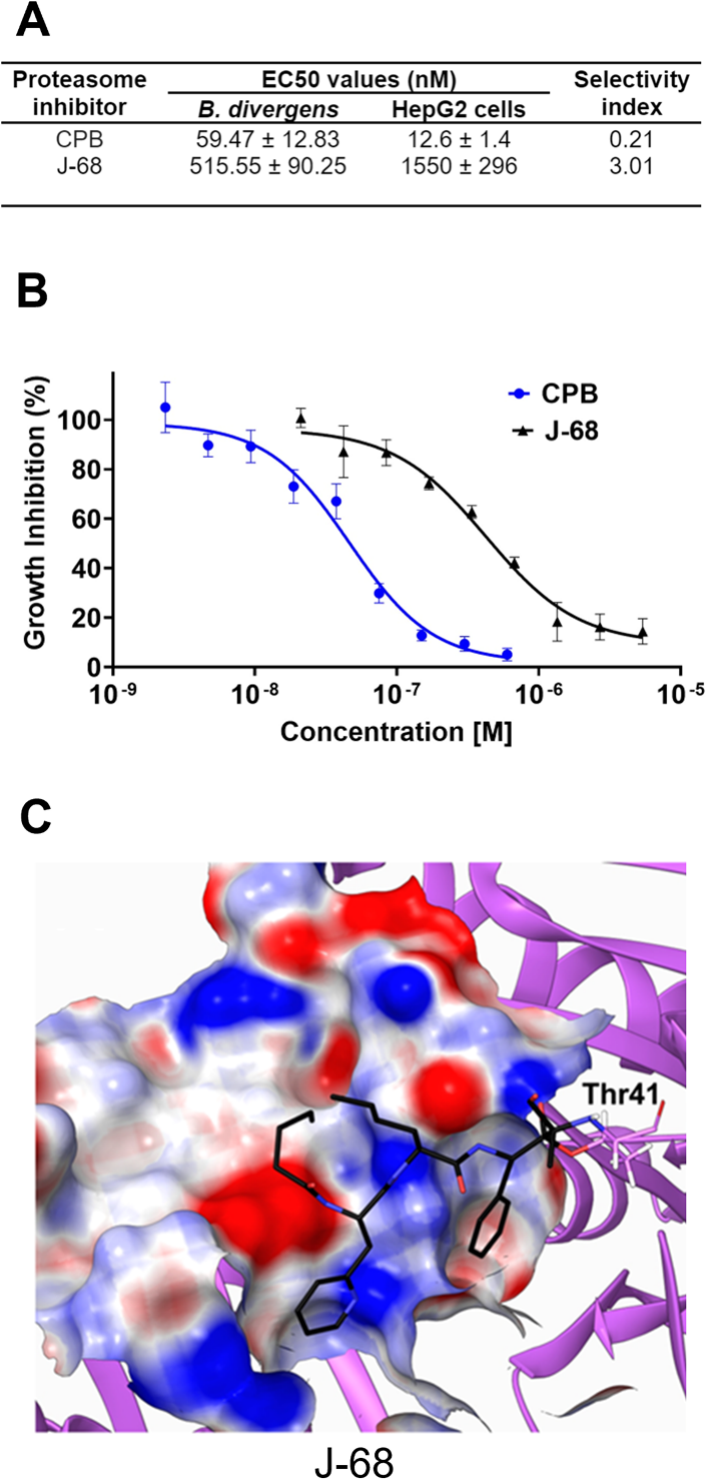
Carmaphycin B-derived compound that is most selective for cultured *B. divergens*. (A) Summary of the EC_50_ values of compounds in *B. divergens* culture and HepG2 cells as well as their respective selectivity index. (B) Dose–response curves for CPB and J-68 with *B. divergens*. (C) In silico visualization of binding of J-68 compound to the *B. divergens* 20S β5 proteasome subunit.

All compounds depicted in Figures 3 and 4 previously underwent evaluation for toxicity in HepG2 cells and the EC_50_ values have been previously reported.^28,27^ Among these, J-68 of carmaphycin B derivatives emerged as the most balanced in terms of high efficacy in activity assays and low HepG2 cell toxicity (Figure 6A). The susceptibility of parasites to J-68 and carmaphycin B^27^ was assessed in *B. divergens*-infected bovine RBC cultures. Parasitemia levels were evaluated using ethidium homodimer labeling and flow cytometry, following the previously described methodology^31^ (Figure 6B and Supplementary Figure 2). Notably, J-68 demonstrated an improved selectivity index over the parental carmaphycin B by 15-fold. However, J-68 is only 3 times more selective for *B. divergens* over HepG2 cells, which is significantly lower than the 2641-fold selectivity that was achieved for the best *P. falciparum* inhibitor. However, that remarkable selectivity was only achieved after iterative synthesis of ∼80 compounds, many of which incorporate of a non-natural d-amino acid at P3 that is favored by *P. falciparum*.^30^ Notably, we also tested compound CP-17^28^ (Supplementary Figure 2) that previously showed significant activity against trichomoniasis^25^ and schistomiasis,^26^ but this compound exhibited a relatively weak effect on RBC-cultured *B. divergens* with EC_50_ of 18.37 ± 7.78 μM.

### 20S Proteasome of *B. divergens* Enrichment by Chromatography

**Figure 7:**
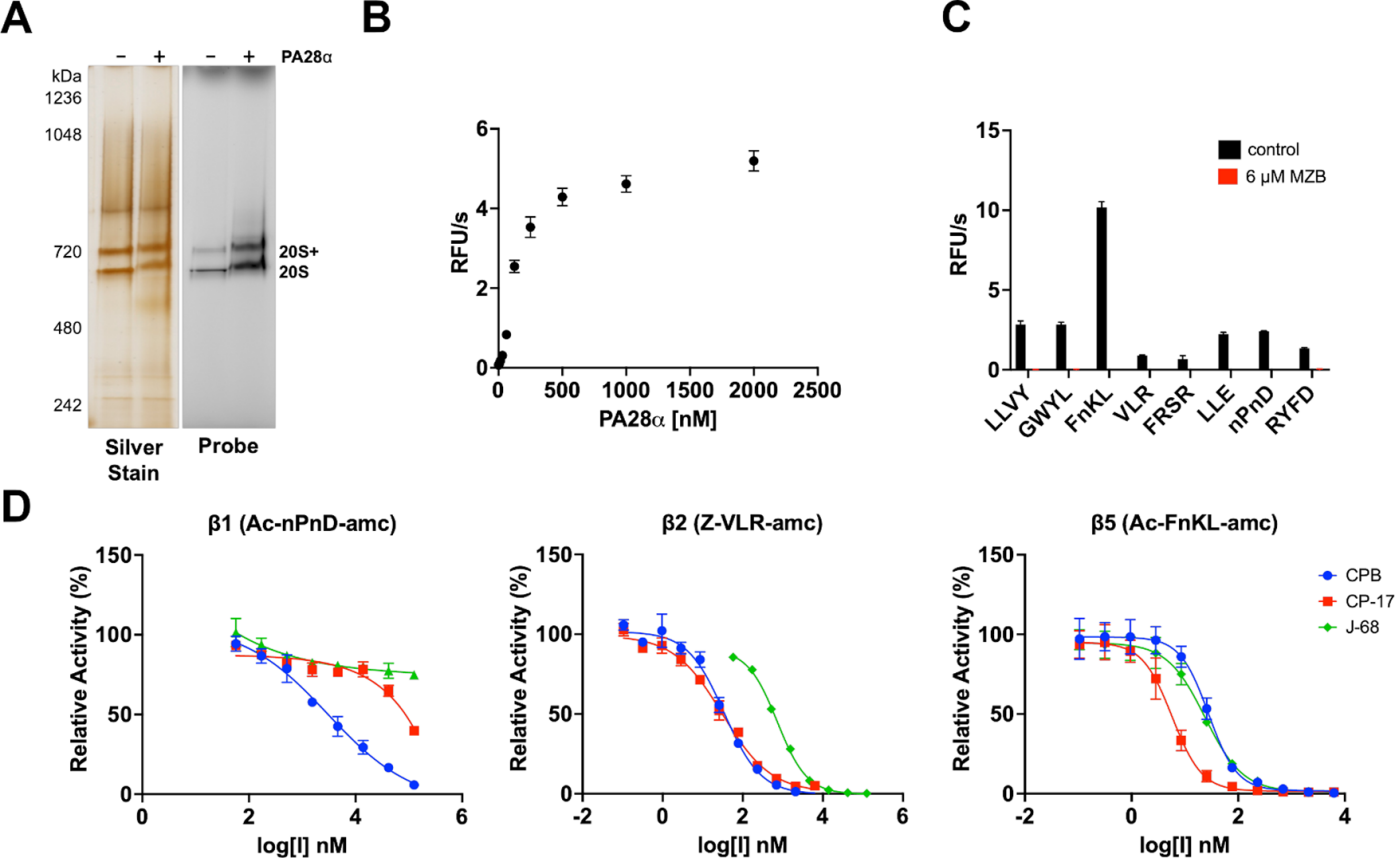
Purification of *B. divergens* proteasome and IC_50_ determination of β1, β2, and β5 activity with selected proteasome inhibitors. (A) Visualization of Bd20S on a native gel in the presence and absence of PA28α. Left gel shows the purity and molecular weight. Right gel shows the active site labeling using Me4BodipyFL-Ahx3Leu3VS. (B) Enzyme activity of Bd20S in the presence of the PA28α activator using the Suc-LLVY-amc substrate. (C) Screen of fluorogenic substrates using Bd20S/PA28α in the presence and absence of 6 μM MZB. DMSO (0.5%) was used as a control. (D) Dose–response curves showing the potency of CPB, CP-17, and J-68 against the β1, β2, and β5 subunits of Bd20S.

To enzymatically characterize the *B. divergens* proteasome, we enriched it from the parasite cell lysate by a three-step fractionation protocol consisting of ultracentrifugation and ion exchange chromatography followed by gel filtration chromatography. All elution fractions were evaluated for activity using the β5 substrate Suc-LLVY-amc in the presence and absence of carfilzomib. While activity was detectable in the fractions, the low level of total protein was evident by optical density measurements at 280 nm. The enriched 20S proteasome was then visualized via silver-stained native PAGE (Figure 7A). To confirm that this protein band was Bd20S, we excised it from the gel for proteomic identity confirmation.

As J-68 is our current lead compound, it was important to understand the mechanism of action. To achieve this, we determined the optimum concentration of PA28α in assays with the isolated proteosome. We show that PA28α increases activity linearly between 10 and 500 nM concentrations in the assay reaction (Figure 7B). Therefore, we decided to use 250 nM for our downstream assays. First, we screened a panel of fluorogenic peptides to find a β5 substrate that is cleaved with higher efficiency than Suc-LLVY-amc (Figure 7C). We determined that Ac-FnKL-amc cleaved with 4-fold higher velocity than Suc-LLVY-amc where lowercase n corresponds to the non-natural amino acid norleucine. This reporter peptide was developed by our group as a substrate for *S. mansoni* 20S proteasome.^43^ We also tested a selection of substrates that were developed to detect β2 activity (Z-VLR-amc and Ac-FRSR-amc) and β1 activity (Ac-nPnD-amc and Ac-RYFD-amc) and found that Z-VLR-amc and Ac-nPnD-amc were the best substrates to use for β2 and β1, respectively. Important, activity with all these substrates was eliminated in the presence of marizomib (MZB), a broad-spectrum proteasome inhibitor. This confirmed that the activity detected from the enriched proteasome sample was not due to a contaminating protease that copurified with Bd20S. Armed with three new substrates, we then performed dose–response assays with CPB and J-68 (Figure 7D). Our studies reveal the CPB inactivates all three subunits but with different potency. The IC_50_ values for β1, β2, and β5 are >3000, 34.29 ± 3.59, and 27.86 ± 2.44 nM, respectively. In comparison, J-68 partially inhibited β1 and had IC_50_ values of 725.2 ± 26.0 and 23.92 ± 2.36 nM for β2 and β5, respectively. In silico analysis of J-68 binding to the *B. divergens* 20S β5 proteasome subunits, as shown in Figure 6C, suggests potential for further docking and optimization to enhance the selectivity of carmaphycin B-based compounds. This potential is further supported by the IC_50_ value of J-68, which confirms its inhibitory efficacy in assays using the purified *B. divergens* 20S proteasome (Figure 7).

## Conclusions

In this study, we have showcased carmaphycin B as a foundational compound with significant potential for the development of innovative chemotherapy options for babesiosis, leveraging its selective inhibition of the parasite over host proteasome. Our exploration across two generations of carmaphycin B derivatives has underscored the compound’s promise and the feasibility of achieving an improved selectivity index. Nevertheless, the outcomes achieved thus far fall short of the benchmarks set by therapies targeting the malaria parasite *P. falciparum*, indicating a need for further empirical inquiry. Future research directions will encompass comprehensive biochemical and structural characterizations of the 20S proteasome derived from *Babesia* species, aiming to delineate the substrate specificity of its catalytic subunits in greater detail. Armed with this enhanced understanding, the next phase of our work will concentrate on the meticulous optimization of these derivatives to augment both their selectivity and therapeutic indices. Our ultimate objective is to contribute to the arsenal of safer, more efficacious treatment modalities for babesiosis, aligning with the overarching One Health approach to combat vector-borne diseases. This endeavor not only addresses an immediate need within infectious disease therapeutics but also paves the way for a broader application of proteasome inhibitors in treating other parasitic infections.

## Supporting Information Available

Supplementary Figure 1: multiple alignments of human activator subunits and their homologs from protozoan parasites; Supplementary Figure 2: treatment of *B. divergens* in bovine erythrocyte cultures with carmaphycin B (CPB) and J-68; Supplementary Figure 3: human PA28α purification; Supplementary Table 1: α and β subunits of 20S proteasome identified in *B. microti* and *B. divergens* clustering with their bovine, human, and *P. falciparum* analogs (PDF)
