# Supplementary material for: Evaluating Antimalarial Proteasome Inhibitors for Efficacy in Babesia Blood Stage Cultures
Source: ACS Omega. 2024 Oct 28;9(45):44989–99. doi: 10.1021/acsomega.4c04564 (PMC11561622; doi:10.1021/acsomega.4c04564)
Supplement: Supplementary file 1 — ao4c04564_si_001.pdf [file ao4c04564_si_001.pdf]

Evaluating antimalarial proteasome inhibitors for efficacy in *Babesia* blood stage cultures.

Luïse Robbertse [a], Pavla Fajtová [c,d], Pavla Šnebergerová [a,b], Marie Jalovecká [a,b], Viktoriya Levytska [a], Elany Barbosa da Silva [c], Vandna Sharma [c], Petr Pachl [d], Jehad Almaliti [e], Momen Al-Hindy [e], William H. Gerwick [e], Evžen Bouřa [d], Anthony J. O'Donoghue [c*], Daniel Sojka [a*]

[a] Institute of Parasitology, Biology Centre of the Czech Academy of Sciences, Ceske Budejovice, Czech Republic

[b] Faculty of Science, University of South Bohemia, Ceske Budejovice, Czech Republic

[c] Skaggs School of Pharmacy and Pharmaceutical Sciences, University of California, San Diego, La Jolla, USA

[d] Institute of Organic Chemistry and Biochemistry, Academy of Sciences of the Czech Republic, Prague, Czech Republic

[e] Center for Marine Biotechnology and Biomedicine, Scripps Institution of Oceanography, University of California San Diego, La Jolla, USA

Corresponding authors: Daniel Sojka (sojkadan@gmail.com); Anthony J. O'Donoghue (ajodonoghue@ucsd.edu)

Supplementary files:

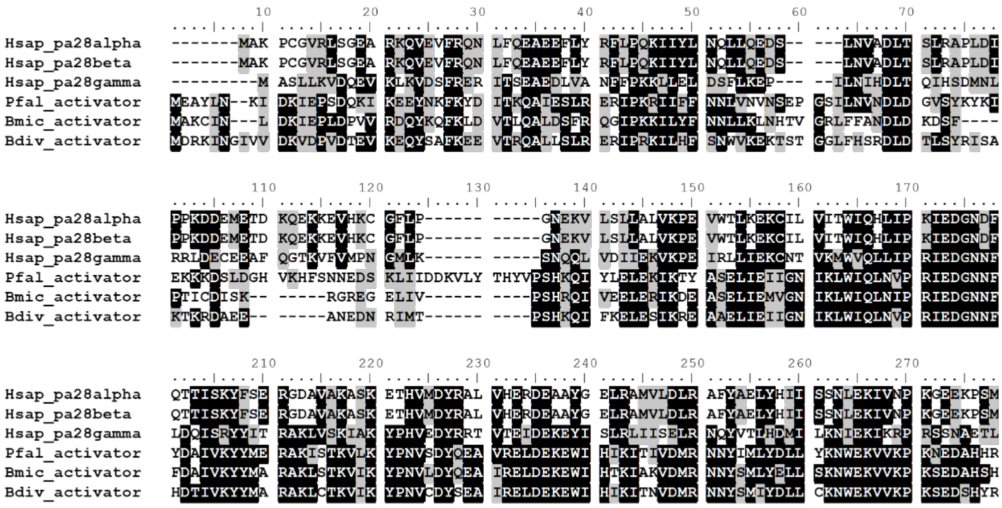

Alignment scores

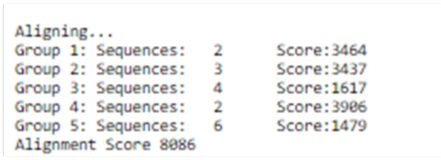

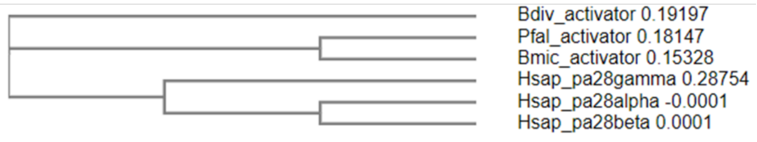

Supplementary figure 1: Multiple alignments of human activator subunits and their homologues from protozoan parasites. Alignment was prepared using Clustal Omega and edited using BioEdit Sequence Alignment Editor. Depicted are three human activators: pa28α (**Uniprot ID: Q9UL46**); pa28β (**GenBank no. NP_058960**) and pa28γ (**GenBank no. P61289**). Additionally, activators from *P. falciparum* (**Uniprot ID: Q8I374**); *B. microti* (**Uniprot ID: I7IFU6**) and *B. divergens* (**PiroplasmBD: Bdiv_002540c-t42_1**) are shown.

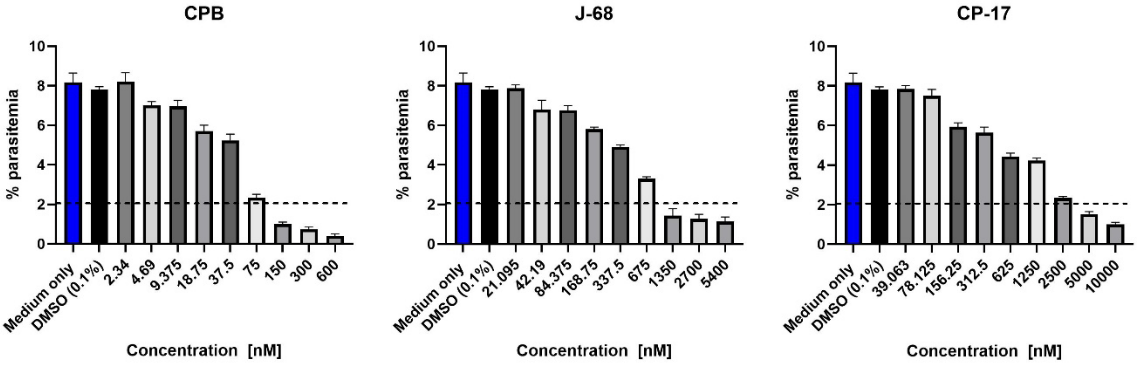

Supplementary figure 2: Treatment of *B. divergens* in RBC *ex vivo* cultures with Carmaphycin B (CPB), J-68 and CP-17. Data represent means of three technical replicates and the error bars indicate standard deviations. Experimental conditions: starting parasitemia 2% (dotted line), medium with inhibitory compounds exchanged in 12 h intervals, total cultivation duration 48 h.

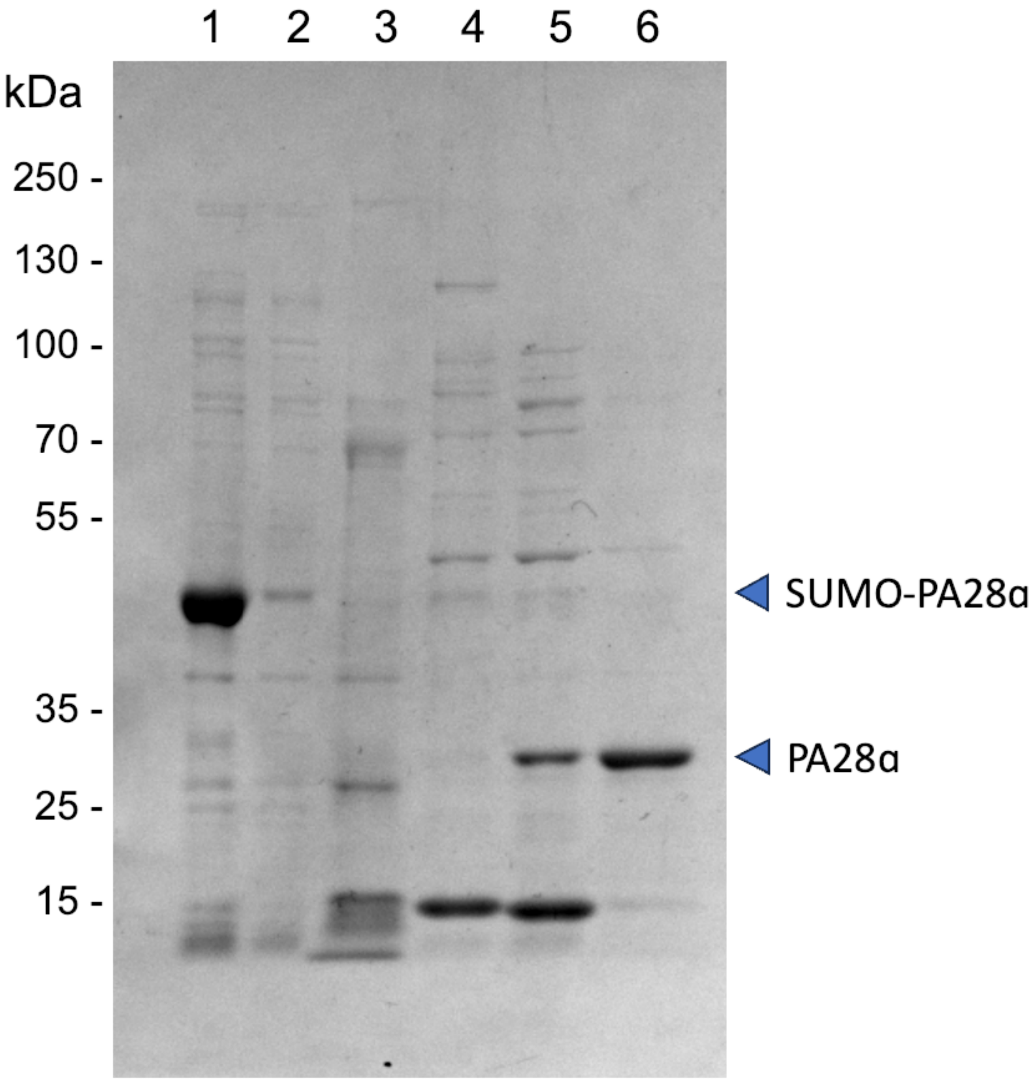

Supplementary figure 3: Human PA28α purification. Protein analysis on SDS denatured gel during PA28α purification: line 1) bacteria lysate after ON PA28α expression, lines 2-4) NiNTa affinity chromatography: 2) flowthrough, 3) column wash with 20 mM imidazole, 4) elution with 250 mM, 5) SUMO-PA28α after sumo protease incubation, 6) purified PA28α (28 kDa) after reverse NiNTA purification and concentration.

Supplementary Table 1

List of organisms and corresponding protein codes used from various databases for the construction of the phylogenetic tree.

subunit	Species	Accession Number Uniprot	Accession Number PiroplasmaDB
α1	*B. divergens*		Bdiv_010920-t42_1
	B. microti	I7IF64	
	B. taurus	Q2YDE4	
	H. sapiens	P60900	
	P. falciparum	Q8IAR3	
α2	*B. divergens*		Bdiv_018040c-t42_1
	B. microti	I7IT56	
	B. taurus	Q3T0Y5	
	H. sapiens	P25787	
	P. falciparum	C6KST3	
α3	*B. divergens*		Bdiv_032250-t42_1
	B. microti	A0A1N6LXS4	
	B. taurus	Q3ZCK9	
	H. sapiens	P25789	
	P. falciparum	Q8IDG3	
α4	*B. divergens*		Bdiv_032240c-t42_1
	B. microti	A0A1N6LXS8	
	B. taurus	Q3ZBG0	
	H. sapiens	O14818	
	P. falciparum	Q8IDG2	
α5	*B. divergens*		Bdiv_003150c-t42_1
	B. microti	I7IS03	
	B. taurus	Q5E987	
	H. sapiens	P28066	
	P. falciparum	Q8IBI3	
α6	*B. divergens*		Bdiv_012740c-t42_1
	B. microti	I7IHE6	
	B. taurus	Q3T0X5	
	H. sapiens	P25786	
	P. falciparum	Q8IK90	
α7	*B. divergens*		Bdiv_006390c-t42_1
	B. microti	A0A1R4ABW5	
	B. taurus	Q58DU5	
	H. sapiens	P25788	
	P. falciparum	O77396	

subunit	Species	Accession Number Uniprot	Accession Number PiroplasmaDB
β1	*B. divergens*		Bdiv_003880c-t42_1
	B. microti	I7IGE2	
	B. taurus	Q3MHN0	
	H. sapiens	P28072	
	P. falciparum	Q8I0U7	
β1i	*B. taurus*	Q3SZC2	
	H. sapiens	P28065	
β2	*B. divergens*		Bdiv_021310-t42_1
	B. microti	A0A0K3APP5	
	B. taurus	Q2TBP0	
	H. sapiens	Q99436	
	P. falciparum	Q8I6T3	
β2i	*B. taurus*	Q3T0T1	
	H. sapiens	P40306	
β3	*B. divergens*		Bdiv_034420c-t42_1
	B. microti	I7JCV5	
	B. taurus	P33672	
	H. sapiens	P49720	
	P. falciparum	Q8I261	
β4	*B. divergens*		Bdiv_014050c-t42_1
	B. microti	I7I8K2	
	B. taurus	Q5E9K0	
	H. sapiens	P49721	
	P. falciparum	Q8IKC9	
β5	*B. divergens*		Bdiv_021660-t42_1
	B. microti	A0A1R4ACG2	
	B. taurus	Q32KL2	
	H. sapiens	P28074	
	P. falciparum	Q8IJT1	
β5i	*B. taurus*	Q3T112	
	H. sapiens	P28062	
β6	*B. divergens*		Bdiv_010570c-t42_1
	B. microti	I7I9Z8	
	B. taurus	Q2TBX6	
	H. sapiens	P20618	
	P. falciparum	A0A5K1K7U1	
β7	*B. divergens*		Bdiv_019340c-t42_1
	B. microti	A0A1N6LWR9	
	B. taurus	Q3T108	
	H. sapiens	P28070	
	P. falciparum	Q7K6A9	
